# Structured illumination of the interface between centriole and peri-centriolar material

**DOI:** 10.1098/rsob.120104

**Published:** 2012-08

**Authors:** Jingyan Fu, David M. Glover

**Affiliations:** Cancer Research UK Cell Cycle Genetics Group, Department of Genetics, University of Cambridge, Cambridge CB2 3EH, UK

**Keywords:** super resolution microscopy, centriole, pericentriolar material, *Drosophila*

## Abstract

The increase in centrosome size in mitosis was described over a century ago, and yet it is poorly understood how centrioles, which lie at the core of centrosomes, organize the pericentriolar material (PCM) in this process. Now, structured illumination microscopy reveals in *Drosophila* that, before clouds of PCM appear, its proteins are closely associated with interphase centrioles in two tube-like layers: an inner layer occupied by centriolar microtubules, Sas-4, Spd-2 and Polo kinase; and an outer layer comprising Pericentrin-like protein (Dplp), Asterless (Asl) and Plk4 kinase. Centrosomin (Cnn) and γ-tubulin associate with this outer tube in G2 cells and, upon mitotic entry, Polo activity is required to recruit them together with Spd-2 into PCM clouds. Cnn is required for Spd-2 to expand into the PCM during this maturation process but can itself contribute to PCM independently of Spd-2. By contrast, the centrioles of spermatocytes elongate from a pre-existing proximal unit during the G2 preceding meiosis. Sas-4 is restricted to the microtubule-associated, inner cylinder and Dplp and Cnn to the outer cylinder of this proximal part. γ-Tubulin and Asl associate with the outer cylinder and Spd-2 with the inner cylinder throughout the entire G2 centriole. Although they occupy different spatial compartments on the G2 centriole, Cnn, Spd-2 and γ-tubulin become diminished at the centriole upon entry into meiosis to become part of PCM clouds.

## 2. Introduction

Centrosomes were first described independently by van Benenden and Boveri in the late nineteenth century as the organelles at the poles of the mitotic spindle [1]. The presence of multiple centrosomes and spindle defects has been known in tumour cells for an almost equally long time, and this led Boveri to his famous work relating the origins of cancer to the aneuploidy arising from such spindle defects [2]. In his detailed descriptions of the centrosome, Boveri noted its increase in size during mitosis [3]. But despite its long history, the roles of the centrosome in cancer and its maturation cycle have remained poorly understood.

The centrosome comprises a pair of centrioles linked together at their proximal regions and surrounded by an amorphous matrix of proteins, the pericentriolar material (PCM), that increases conspicuously in size upon mitotic entry [4–8]. Centrioles also act as basal bodies to template the growth of cilia and flagella [9,10], and are dispensable parts of the centrosome in female meiosis in many metazoans [11–13]. When present, however, centrioles appear to organize the PCM [14].

Several centriolar proteins have ambiguous functions both at the centriole and in the PCM. Spd-2, for example, lies on top of the hierarchy of centriole assembly in *Caenorhabditis elegans,* where it is required for the activity of the Zyg-1 protein kinase [15–19]. In *Drosophila*, on the other hand, Spd-2 plays no role in centriole assembly but is required for the recruitment of PCM upon mitotic entry [20,21]. Here we ask whether understanding of the roles of this and other centriole/centrosome proteins can be furthered by better knowledge of their spatial relationships throughout centrosome maturation using *Drosophila* as a model organism.

A different protein kinase, Polo-like kinase 4 (Plk4), lies at the head of the pathway for centriole assembly in *Drosophila* and human cells [22–24]. The centriolar protein Asterless (Asl; *Drosophila*)/Cep152 (human) serves as a scaffold for Plk4 recruitment in order to permit centriole assembly [25–27]. The Zyg-1 or Plk4 protein kinases are required to recruit Sas-5 (*C. elegans*)/Ana2 (*Drosophila melanogaster*)/Stil (human) and Sas-6 [18,19,28–34]. In most centrioles, a cartwheel structure is assembled at the very proximal part and in the newly forming procentriole to establish centriole symmetry. Loss of Sas-6 in *Chlamydomonas*, *Paramecium* and *Drosophila* results in a reduction in numbers of centrioles and the centrioles that do form have missing microtubules [35–37]. By contrast, co-expression of Sas-6 with Ana2 in *Drosophila* spermatocytes leads to the assembly of cartwheel-like structures [31]. Sas-6 has been shown to assemble into stable structures [38], and recent structural analyses of Sas-6 have provided a physical explanation for the nine-fold symmetry of the cartwheel [39,40].

Recruitment of Sas-4 onto the nine-fold symmetrical structure takes place downstream of Sas-5/Sas-6 [18,19], and is required for the incorporation of singlet microtubules in a γ-tubulin-mediated process [41]. Sas-4 helps to promote the polymerization of centriolar microtubules in the direction of the distal end, and overexpression of its human homologue, CPAP, leads to the formation of elongated centrioles [42–44]. A capping protein, CP110, appears to limit this lengthening in HeLa and U2OS cells, where its depletion also leads to abnormally long centrioles [43]. In cultured *Drosophila* cells, however, depletion of the Cp110 capping protein leads to the shortening of centriole microtubules apparently by exposing them to the microtubule depolymerizing Klp10A protein [45]. CP110 together with Cep97 also play a role in regulating formation of primary cilia; in RPE1 cells, their depletion promotes whereas their overexpression prevents primary cilia formation [46,47]. How the length of centrioles becomes fixed and how this can vary between different tissues of the same organism is poorly understood. *Drosophila* spermatocytes present an excellent model in which to examine this process as their centrioles become highly elongated.

Sas-4 is a cornerstone for protein–protein interactions, and it is of particular interest to understand its spatial relationships to its partners. Sas-4 can physically interact with α/β-tubulin dimers and with Sas-5/Ana2/Stil, suggesting it physically couples the cartwheel to microtubules [32,48,49]. It also physically interacts with Asl/Cep152, and expression in *Drosophila* embryos either of Sas-4 alone or a mutant form of Asl able to bind Sas-4 but not Plk4 leads to the formation of acentriolar PCM aggregates able to nucleate cytoplasmic microtubules [26]. Further evidence for the involvement of Sas-4 in PCM recruitment comes from its ability to form complexes with Centrosomin (Cnn), Asl and Pericentrin-like protein (Dplp), and the finding that centrosomes with mutant Sas-4 unable to form such complexes have reduced PCM [26,50]. Injection of antibodies against Asl or Spd-2 into *Drosophila* embryos prevents recruitment of Cnn that normally occurs in the vicinity of the centriole [51], providing further support for the participation of these molecules in the maturation process. This also accords with an earlier finding by Varmark *et al*. [52] of reduced amounts of PCM in *asl* mutants.

Another Polo-like kinase, Plk1 or Polo in *Drosophila*, is required for the dramatic increase in PCM that occurs prior to mitotic entry [53,54]. Of the proteins recruited by Plk1, the γ-tubulin ring complex is of primary significance, as this will lead to increased nucleation of cytoplasmic microtubules that is necessary to form the spindle [55]. In human cells, the levels of Cep192/hSpd-2, Pericentrin and Cep215/Cdk5Rap2/Cnn all increase upon centrosome maturation, and these proteins appear to be interdependent for the recruitment of γ-tubulin [56,57]. Two genome-wide siRNA screens designed specifically to identify *Drosophila* genes required for centrosome duplication and maturation identified the fly counterparts of these three proteins, together with Polo kinase and the PP2A phosphatase [58,59]. In *C. elegans*, Spd-2 has been shown to bind to and so recruit Polo kinase and thereby provide a limiting factor for total centrosome size [60]. The involvement of Spd-2 in centrosome maturation in *Drosophila* is seen by the compromised recruitment of PCM in both Spd-2 mutant somatic cells and in spermatocytes, where it was also shown to be required for cohesion between the two centrioles [20,21].

Although not essential for many aspects of centrosome function [61], centrioles do facilitate the nucleation of PCM, and several of the centriolar proteins do appear to contribute to PCM recruitment, indicating the coevolution of these structures. Moreover, in their screens for proteins required for centriole duplication and centrosome maturation, Dobbelaere and colleagues [59] identified several centriolar proteins apparently required for both processes. How can such bifunctionality of centriolar proteins relate to their physical organization? Because the centrosome is close in size to the diffraction limit for conventional microscopy, immuno-electron microscopy (EM) was previously the only means of addressing this question. While this approach has been invaluable, difficulties arise from preserving antigens throughout fixation, and the discontinuous gold signal makes precise positioning of antigens and co-localization analysis very difficult. To overcome these limitations, we have applied three-dimensional-structured illumination microscopy (3D-SIM) to analyse the spatial inter-relationships between centriolar and pericentriolar proteins in *Drosophila* as centrioles grow and as the centrosome matures for its functions in M phase.

## 3. Results and discussion

### 3.1. Mother centrioles accumulate a unique set of proteins

**Figure 1.**
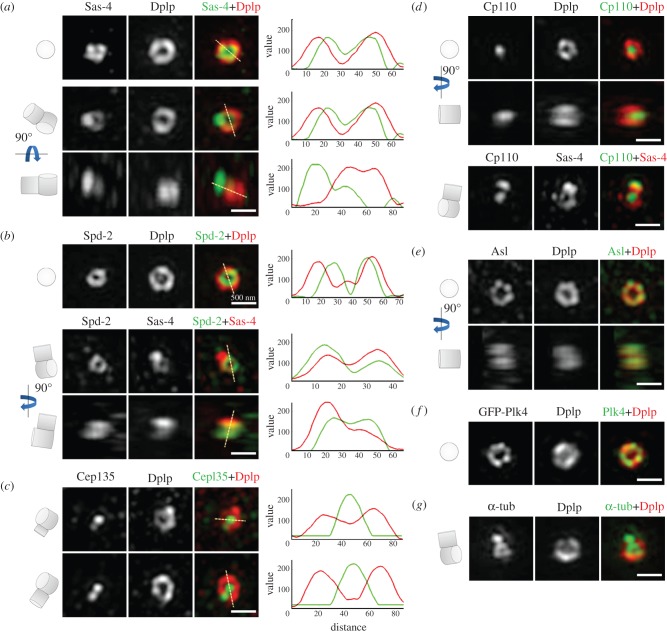
Localization of centriolar proteins in the centrosomes of cultured D.Mel-2 cells. D.Mel-2 cells stained to reveal the indicated centriolar proteins. (*a*) Sas-4 (green) and Dplp (red). The upper row shows a centriole before a procentriole becomes visible. Sas-4 is present in a similar cylindrical structure as Dplp but of a smaller diameter. As the daughter centriole elongates, it is loaded with Sas-4 (middle and bottom rows), whereas Dplp only decorates the mother centrioles. (*b*) Spd-2 (green) and Dplp (red), or Spd-2 (green) and Sas-4 (red). Spd-2 is present in a cylinder within the Dplp (upper row) and similar in diameter to the Sas-4 cylinder. Sas-4 is present on mother and daughter centrioles while Spd-2 predominantly on the mother centriole (middle and bottom rows). (*c*) Cep135 (green) and Dplp (red). Cep135 is present in the innermost part of mother centrioles and recruited to procentrioles in levels reflecting elongation. By contrast, Dplp preferentially associates with the mother centrioles, regardless of whether the daughter centrioles fully elongate. (*d*) Cp110 (green) and Dplp (red). Top view shows Cp110 to occupy the central most part of the centriolar cylinder (upper row). Side view shows Cp110 as a plug-like body at the distal end of a single centriole (middle row). Staining to reveal Cp110 (green) and Sas-4 (red) shows that Cp110 is present at the distal end of both mother and daughter centrioles (bottom row). (*e*) Asl (green) and Dplp (red) showing their overlapping distribution in cylindrical structures. Asl shows a punctate staining pattern within the nine-fold symmetrical structure. (*f*) D.Mel-2 cells expressing GFP-Plk4 (green) and stained to reveal Dplp (red). The distribution of Plk4 strongly resembles that of its partner protein Asl. (*g*) D.Mel-2 cells stained to reveal α-tubulin (α-tub; green) and Dplp (red). α-tubulin is present in a cylinder that lies within the region of Dplp staining and that presumably corresponds to the nine sets of microtubules. It is also seen in the daughter centriole. Scale bars, (*a*–*g*) 500 nm.

**Figure 2.**
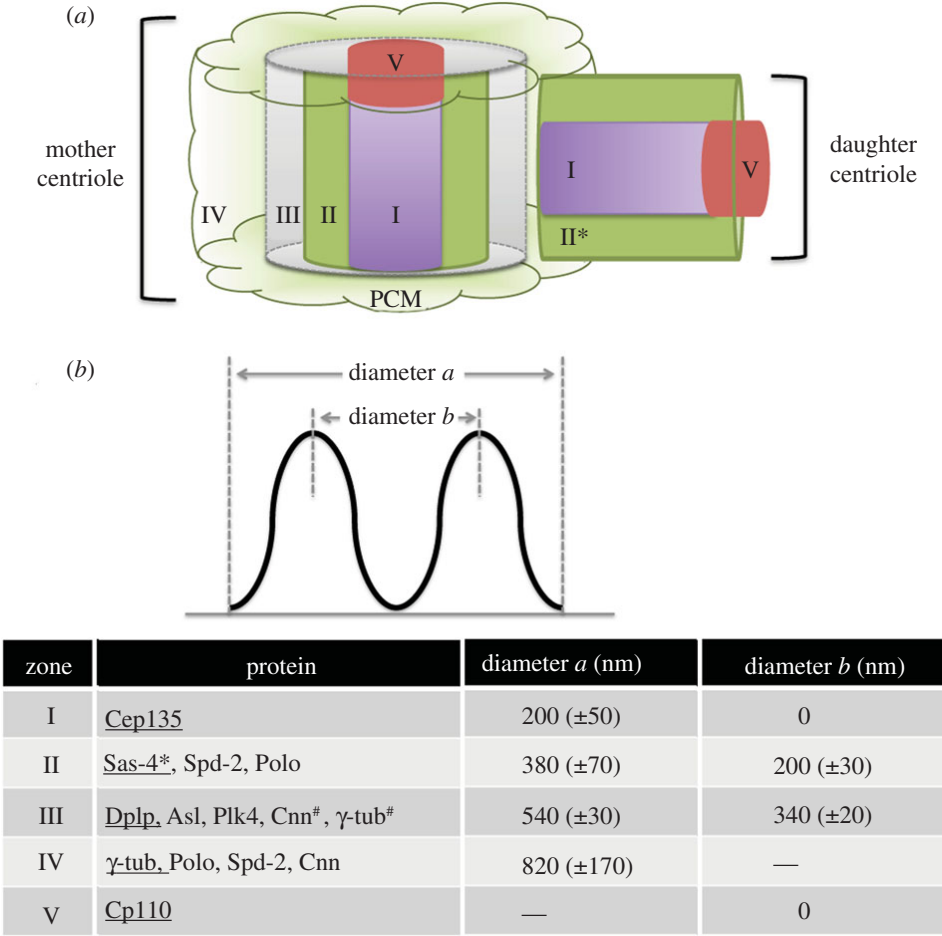
Scheme depicting the structure of a centrosome and the distribution of different proteins. (*a*,*b*) The mother centriole is shown partitioned into five zones corresponding to the zones of staining that we described. Zones III, IV and part of the zone II (proteins without asterisk) are absent from the daughter centriole. Also, parts of the zone III (proteins marked with #) only start to accumulate in late G2 phase. The proteins present in each zone are listed in the table, and the diameters (±s.d.) of the zones are defined by the staining patterns as indicated. tub, tubulin.

In order that we could apply 3D-SIM to study the localization of centrosomal antigens in *Drosophila*, we first needed to select reference molecules for co-immunostaining. We chose two centrosome components: Sas-4, known to bind tubulin dimers and interact with the centriolar microtubules to promote their elongation [42–44,48,49]; and the *Drosophila* protein Dplp, reported to associate tightly with centrioles in interphase [62]. Three-dimensional reconstruction of the interphase centrosome resolved the Sas-4 and Dplp-associated structures into cylinders, with average diameters of 200 and 340 nm, respectively (figures 1*a* and 2). The diameter of this inner tube of Sas-4 is similar to the diameter of the *Drosophila* centriole measured to the doublet microtubules by EM as 180 nm [45]. This suggests that Sas-4 is likely to be associated with the centriolar microtubules in accord with its ability to bind α/β-tubulin as the centriolar microtubules are being built [48,49]. Dplp lies on the outer face of the cylinder marked out by the nine sets of centriole microtubules and is thus positioned to form an interface with the PCM.

In addition to the concentric cylindrical staining given by anti-Sas-4 and -Dplp antibodies, we found a separate very strong focus of additional staining given by anti-Sas-4 but not by anti-Dplp (figure 1*a*). We also saw a similar localization of green fluorescent protein (GFP) in cells expressing Sas-4-GFP (data not shown), and we interpreted this additional body to be a daughter centriole. To understand this distribution of Sas-4 better, we set out to localize it with respect to Spd-2, which we found also to occupy a cylinder of diameter 200 nm lying within and concentric to the Dplp cylinder and thus broadly coincident with the Sas-4 cylinder (figure 1*b*). Once again, we were able to detect an additional strong block of Sas-4 apparently orthogonal to the Sas-4 cylinder in the position of a daughter centriole (figure 1*b*). Spd-2, on the other hand, was restricted to the larger centriole as we had found for Dplp, suggesting that these two proteins might only be recruited to the mature, mother centriole.

The proteins required to initiate formation of the cartwheel should also be associated with procentriole and have a similar localization to Sas-4. To test this, we co-stained the cells with Dplp and Cep135—the orthologue of Bld10p that has been shown to constitute the tip of the cartwheel spokes in *Chlamydomonas* [63]. Bld10p is required for cartwheel formation in *Chlamydomonas* and *Paramecium* [37,63], and Cep135 is required for excessive centriole duplication in Plk4-overexpressing human cells [29]. In *Drosophila* when it is absent, centrioles are able to form but are shorter in length [64,65]. We found one punctate body of Cep135 at the centre of the Dplp cylinder and a second punctate body that was not surrounded by Dplp (figure 1*c*). This second body varied in intensity between cells, suggesting different extents of procentriole growth leading to increased incorporation of Cep135. The body at the centre of the Dplp cylinder was of constant intensity, consistent with this being the mature mother centriole. This is in accord with findings that Cep135 can be assembled into early daughter centrioles in spermatocytes [64]. We found that all procentrioles we were able to observe were positive for Cep135 and Sas-4, but lacked Dplp and Spd-2. We also confirmed this localization with mitotic centrosomes that contain mature mother centrioles and fully elongated daughters. Dplp and Spd-2 signals were restricted to the mother centrioles, whereas Sas-4 and Cep135 stained both mothers and daughters.

We then examined the localization of Cp110 that forms a cap on the distal end of centrioles and is required to control centriole length [43,45,46,66]. In accord with this, we found that mature centrioles had Cp110 at the very centre of the Dplp cylinder at the distal end (figure 1*d*). It constituted a plug-like structure extending midway into the distal lumen of the centriole. We then localized Cp110 with respect to Sas-4. The anti-Sas-4 antibody stained the cylindrical mother centriole structure and its orthogonal daughter centriole, and Cp110 was associated with both (figure 1*d*). This would accord with the idea that Cp110 caps the distal end of the centriole microtubules both during and following completion of their growth. Taken together, these results indicate that whereas Cep135, Sas-4 and Cp110 are associated with both mother and daughter centrioles, Dplp and Spd-2 are only present on the mother centriole.

**Figure 3.**
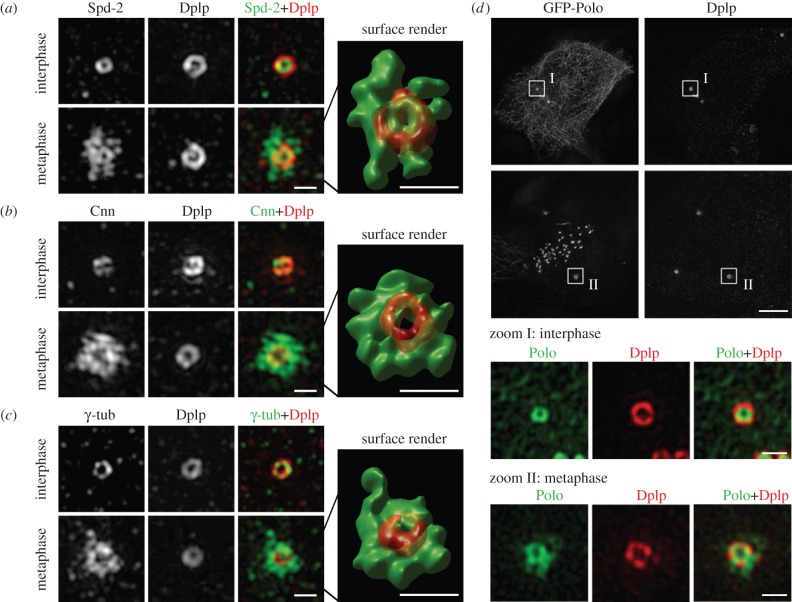
Development of the pericentriolar material (PCM) during centrosome maturation. (*a*–*c*) D.Mel-2 cells were stained to reveal (*a*) Spd-2 (green) and Dplp (red), (*b*) Cnn (green) and Dplp (red), or (*c*) γ-tubulin (green) and Dplp (red). Representative interphase and metaphase centrioles are shown. Spd-2 localizes to all interphase centrioles, whereas Cnn and γ-tubulin are found only on a small subset of interphase centrioles. All three proteins display cylindrical structures in interphase with the diameters of Cnn and γ-tubulin cylinders being similar to those of Dplp. Spd-2, on the other hand, is more compact around the centriole core. During mitosis, there is a large accumulation of pericentriolar Spd-2, Cnn and γ-tubulin with only Spd-2 being still detectable at the core of the centriole itself. Scale bars: 500 nm. (*d*) D.Mel-2 cells expressing GFP-Polo were stained to reveal Dplp. Centrosomes from interphase (I) and metaphase (II) cells are shown. In interphase cells, Polo is present both in centrioles as a cylinder within the Dplp cylinder and also on cytoplasmic microtubules. In metaphase, Polo extends into the PCM but not as extensively as Spd-2, Cnn and γ-tubulin. Scale bar in upper row: 4 μm; lower rows: 500 nm.

The ability of centrioles to be formed de novo in unfertilized *Drosophila* eggs following the expression of either Plk4 or its binding partner Asl previously led us to postulate that the role of the mother centriole in centriole duplication is to provide a platform that concentrates Plk4 to direct the seeding of the formation of procentrioles [24–27]. Thus, we asked whether Asl might have some specific distribution on centrioles. We found that the distributions of Asl and Dplp overlapped within a similar cylindrical space. They often showed some particulate substructure, suggesting that they might be closely associated with the individual microtubules (figure 1*e*). We found that Plk4 showed a very similar distribution (figure 1*f*) in accord with the two proteins existing in a complex [25–27]. Asl and Plk4 were present on the walls of this tube on the outer-most part of the centriolar cylinder in all interphase cells. This was in contrast to Cnn and γ-tubulin, which also showed co-localization with Dplp but only in a small portion of interphase cells that were likely to be in late G2 phase, indicative of the first recruitment of PCM prior to mitosis (figure 3*b,c*).

Together, our findings suggest that several proteins implicated as having some role in PCM recruitment at mitotic entry are organized within discrete outer zones of the centriole (figure 2). We define the inner lumen of the centriole occupied by Cep135 as zone I and this constitutes a central cylindrical block extending in its diameter to approximately 200 nm. Sas-4 and Spd-2 both lie in the adjacent zone II, forming the walls of a cylinder of diameter 200 nm at their peak intensity corresponding in position to the region also occupied by microtubules. Accordingly, anti-α-tubulin also labelled this same region (figure 1*g*). Outer zone III comprises another cylinder with walls built of Dplp, Asl and Plk4 but also containing Cnn and γ-tubulin from late G2 phase onwards. These findings are broadly in accord with localization studies for the counterparts of those proteins by immuno-EM. These studies placed human and *Drosophila* Cep135 in the lumen of the centriole (zone I) [29,64], CPAP/hSas-4 associated with the outer wall of the microtubules (zone II), Cep152/Asl on the outer-most circumference of this structure (zone III) and CP110 as a distal cap [25,29].

### 3.2. Pericentriolar material is an ordered structure that expands during mitosis

To evaluate changes in spatial organization of centrosomes upon their maturation at mitotic entry, we focused upon molecules implicated in PCM recruitment. The scaffold protein Asl physically interacts with both Plk4 and Sas-4, the latter having been implicated in PCM recruitment [26,50]. We found, however, that both Asl and Sas-4 had similar distributions during interphase and mitosis (data not shown), suggesting that their function might be more one of seeding PCM formation as previously suggested [26,50]. Dplp also retained a similar distribution in mitotic cells to interphase cells (figure 3) and thus contrasts with its mammalian counterpart, pericentrin, which undergoes considerable expansion in the PCM upon mitotic entry [56]. In contrast, Spd-2, Cnn and γ-tubulin each expanded massively upon the onset of mitosis to occupy broadly similar spatial volumes flanking the perimeter of the mother centriole rather than lying on the top or the bottom of the centriole cylinder (figure 3*a–c*). We could still detect a portion of Spd-2 retained upon the mitotic centriole *per se*, lying inside the Dplp cylinder (figure 3*a*). On the other hand, Cnn and γ-tubulin appeared to be mainly present in the PCM (figure 3*b*,*c*). The outer dimensions of γ-tubulin staining reached 820 nm (figure 2*b*).

The presence of Cnn on the centriole later in G2 and its spreading onto the PCM in mitosis would be consistent with a report that Cnn molecules incorporate into the PCM closest to the centrioles and then spread outwards through the rest of the PCM [51]. Moreover, these authors also reported that daughter centrioles in syncytial embryos only start to incorporate Cnn as they disengage from their mothers [51]. This accords with our finding that Dplp, Cnn and γ-tubulin, all constituents of zone III in mother centrioles, were absent from the similar part of daughter centrioles. Moreover, the PCM staining given by anti-Spd-2, -Cnn and -γ-tubulin antibodies was also not distributed around the daughter centriole. This would be consistent with these molecules providing the seeding capacity for PCM formation and, in the case of Spd-2, Cnn and γ-tubulin, the major PCM components present only around the mature centriole.

Recruitment of PCM during centrosome maturation has been shown to depend upon the mitotic kinase Polo/Plk1 [67–69]. Polo/Plk1 is long known to be associated with the mitotic centrosome, but its precise localization with respect to centrioles and PCM has not been clear. In mammalian cells, Plk1 is degraded around telophase [70,71], reinforcing a notion that the enzyme needs to be synthesized de novo each cycle. On the other hand, maternally provided *Drosophila* Polo can persist until the late third larval instar, the typical lethal phase of strong hypomorphic *polo* mutants [67]. Thus, should proteolysis of Polo occur each cycle, it might not necessarily be expected to result in a complete loss of the kinase, at least in *Drosophila* cells. Accordingly, we were able to detect Polo kinase on the centrioles of interphase cells, where it occupied a cylindrical structure within the Dplp cylinder with a distribution being similar to Sas-4 and Spd-2 in zone II (figure 3*d*). During metaphase, we found it extended into the PCM but occupied a space approximately 620 nm in width and thus this was not as widely distributed as Spd-2, Cnn and γ-tubulin (figure 3*d*). Our unpublished studies show that Polo phosphorylates both Spd-2 and Cnn. Thus, its localization suggests that Spd-2 might be its immediate substrate upon mitotic entry and that it may then contribute to PCM assembly by establishing a spatial phosphorylation gradient.

### 3.3. Centrosome maturation depends on Polo, Cnn and Spd-2

**Figure 4.**
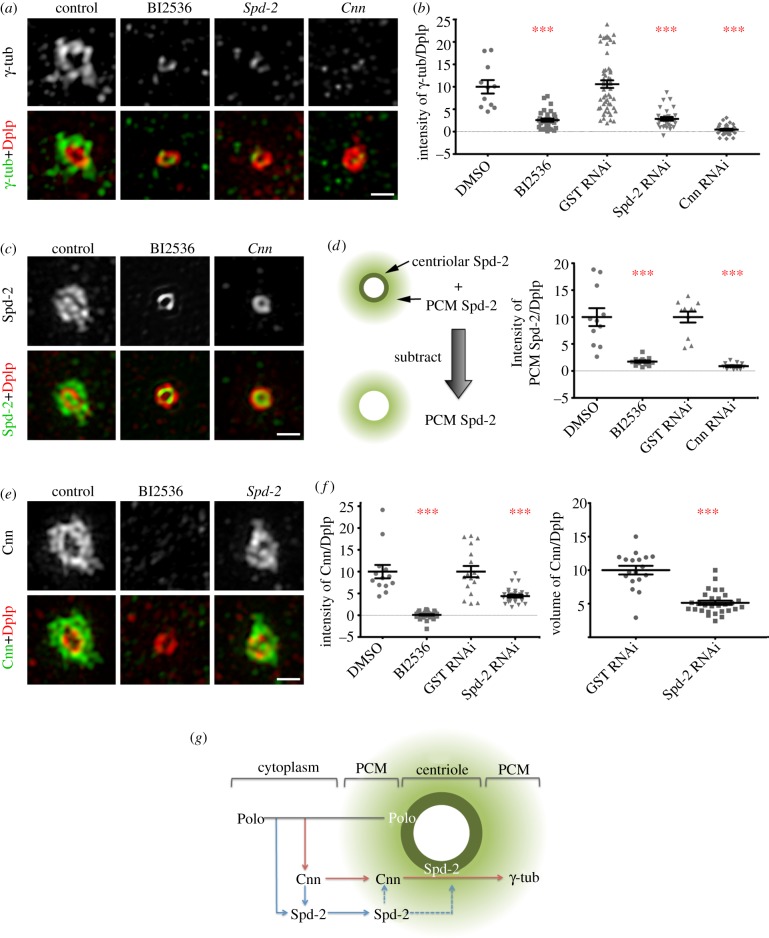
Interdependence of Polo, Cnn and Spd-2 in centrosome maturation. (*a*,*b*) D.Mel-2 cells were treated with dimethylsulfoxide (DMSO) or BI2536 for 16 h, or with glutathione S-transferase (GST), Spd-2 or Cnn dsRNA, and stained to reveal γ-tubulin (green), Dplp (red) and phospho-Histone H3 Ser10 (not shown). Note that in following inhibition of Polo or depletion of Spd-2 or Cnn, γ-tubulin is removed from PCM and only a remnant remains on the centriole. Quantitation of fluorescence intensity of γ-tubulin is normalized to that of Dplp. Error bars represent s.e.m. and asterisks indicate *p* < 0.0001. (*c*,*d*) D.Mel-2 cells were treated with DMSO or BI2536 for 16 h, or with GST or Cnn dsRNA, and stained to reveal Spd-2 (green), Dplp (red) and phospho-Histone H3 Ser10 (not shown). Note that both Polo inhibition and Cnn depletion completely remove Spd-2 from PCM, but the inner cylindrical structure of Spd-2 in the centriole remains intact. Quantitation of total fluorescence intensity of Spd-2 in the pericentriolar region is normalized to that of Dplp. This required subtracting fluorescence of inner cylindrical structures. Error bars represent s.e.m. and asterisks indicate *p* < 0.0001. (*e*,*f*) D.Mel-2 cells were treated with DMSO or BI2536 for 16 h, or with GST or Spd-2 dsRNA and stained to reveal Cnn (green), Dplp (red) and phospho-Histone H3 Ser10 (not shown). Quantitation shows fluorescence intensity of Cnn or total volume occupied by Cnn normalized to that of Dplp. Error bars represent s.e.m. and asterisks indicate *p* < 0.0001. Whereas Cnn is completely removed from the PCM after BI2536 treatment, after Spd-2 depletion the fluorescence intensity of Cnn is reduced by 56% and the volume it occupies by 49%. (*g*) Schematic showing the hierarchy of PCM assembly. Polo localizes to the centriole and extends only a little into the PCM. Spd-2 occupies a similar part of the centriole but is more diffuse in PCM. Cnn and γ-tubulin, on the other hand, are harboured only in PCM, lying outwards of Dplp. During PCM assembly, Polo, that could be either cytoplasmic or centriole-associated, activates Cnn, allowing it to localize to PCM. Both of them are subsequently required for Spd-2 recruitment to the PCM. Cnn cooperates with Spd-2 to recruit γ-tubulin. In addition, Spd-2 is also required to maintain Cnn levels. Scale bars, (*a*,*c*,*e*) 500 nm.

The above findings led us to investigate the interdependencies of Spd-2, Cnn, γ-tubulin and Polo kinase in centrosome maturation. We chose to study this in cultured cells because of the ease whereby RNA interference (RNAi) can be applied to deplete proteins. A major objective of the centrosome maturation process is to accumulate γ-tubulin in order to greatly enhance the nucleation of microtubules during M phase [55]. Thus, we used γ-tubulin staining as the marker to indicate the most downstream event of PCM assembly. We either treated cells with small molecule kinase inhibitors against Polo or Aurora A, or carried out RNAi depletion to deplete individual proteins, including Spd-2, Cnn, Asl, Dplp, Sas-4 and Polo. Of all these treatments, only inhibition/depletion of Polo, Spd-2 and Cnn had significant effects in removing γ-tubulin from PCM. Sas-4 or Asl depletion caused a reduction in the number of centrosomes in the cell, owing to their critical roles in centriole duplication. However, in the cells that retained only one centriole, the recruitment of γ-tubulin was not affected significantly. We presume that the thresholds of Sas-4 or Asl required for centriole duplication are higher than those for the PCM recruitment. These outcomes are consistent with the results of previous genome-wide RNAi screens [58,59]. We found that inhibition of Polo kinase with BI2536 treatment, or depletion of Cnn or Spd-2 virtually eliminated the accumulation of γ-tubulin in the PCM in mitosis (figure 4*a*,*b*). Although both Spd-2 and Cnn RNAi were equally effectively depleted after three rounds of RNAi (data not shown), Cnn depletion was more effective at preventing γ-tubulin recruitment than Spd-2 depletion or indeed than Polo inhibition by BI2536 treatment, suggesting Cnn might be the direct regulator of γ-tubulin recruitment.

We then examined the recruitment of Spd-2 to PCM following Polo kinase inhibition or Cnn depletion. Both treatments resulted in the complete loss of Spd-2 from the PCM without affecting the association of Spd-2 with zone II of the centriole (figure 4*c*,*d*; Spd-2 levels in the PCM were measured after subtracting the centriolar contribution using a circular mask). Thus both Polo activity and Cnn are required to recruit Spd-2 to the PCM. This finding seems at odds with a previous report that described Spd-2 localization at the centrosome to require Asl but not Dplp, Cnn or γ-tubulin [21]. However, this could reflect either differences between cell types or the use of mutants in these studies that were not nulls, or that Spd-2 at the centriole masked any changes in Spd-2 in the PCM.

Finally, we examined Cnn recruitment to the PCM following either Polo inhibition or Spd-2 RNAi. This revealed that when Polo activity was inhibited, Cnn was completely prevented from recruitment to PCM, whereas depletion of Spd-2 reduced the levels of Cnn in PCM only by 56 per cent (measured by fluorescent intensity) or 49 per cent (measured by volume) (figure 4*e*,*f*). Thus, Cnn is dependent upon Polo activity for its localization to the PCM but is only partially dependent upon Spd-2. This is consistent with the previous finding that PCM recruitment in Spd-2 mutant somatic cells is only partially compromised [20].

Together, these results led us to the model presented in figure 4*g*, in which recruitment of γ-tubulin is primarily dependent upon Cnn, which is in turn dependent upon Polo. It is facilitated by Spd-2, whose recruitment to the PCM requires both Polo and Cnn. There are strong parallels between these findings and a study that showed interdependency of Cep192/hSpd-2 and Cep215/Cdk5Rap2/Cnn in the recruitment of the γ-tubulin ring complex in human cells [57]. However, it seems that in the *Drosophila* cells, Spd-2 and Cnn are only partly interdependent as substantial levels of Cnn can be recruited in the absence of Spd-2.

### 3.4. Centriole growth and centrosome maturation in the extended G2 preceding male meiosis

In contrast to somatic cells, the centrosomes of spermatocytes undergo two types of maturation in preparation for male meiosis: first, their centrioles undertake a dramatic lengthening in the extended G2 phase that takes place in concert with the growth in cell size; and second at the onset of male meiosis they develop PCM. We therefore wished to examine how the organization of centrioles might change through the growing phase; how PCM antigens are recruited onto the elongated centrioles; and finally, how PCM antigens are then redistributed upon entry into meiosis.

**Figure 5.**
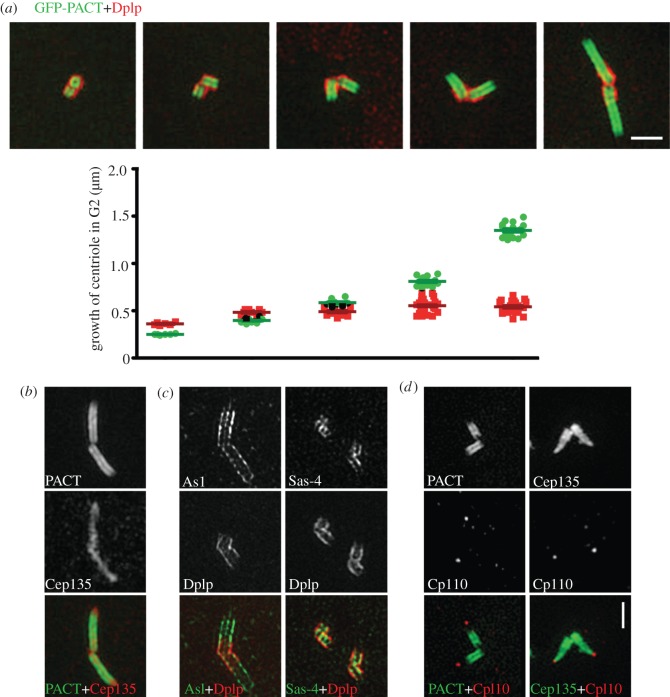
Growth of spermatocyte centrioles in the extended G2 preceding meiosis. (*a*) Centrioles from cysts of primary spermatocytes at progressively later stages of G2 expressing GFP-PACT (green) were stained to reveal Dplp (red). Dplp remains associated with the hinge area and the proximal part of the centriole that increases in length from approximately 360 to 540 nm (red). During the same interval, centriolar microtubules marked with GFP-PACT increase in length from 250 nm to 1.4 μm (green). (*b*) Mature primary spermatocyte centrioles (still in G2) expressing GFP-PACT (green) and stained to reveal Cep135 (red). Note that Cep135 extends down the length of the GFP-PACT cylinder. (*c*) Mature primary spermatocyte centrioles stained to reveal Asl (green) and Dplp (red). Asl extends along the length of the centriole in a cylinder of similar diameter to Dplp, which is restricted to the proximal part; Sas-4 (green) and Dplp (red). Both Sas-4 and Dplp are restricted to the proximal part, with Sas-4 staining being contained within that of Dplp. (*d*) Immature primary spermatocyte centrioles expressing GFP-PACT or GFP-Cep135 (green) were stained to reveal Cp110 (red). Note that Cp110 shows localization at the distal ends separated from GFP-PACT. On the contrary, Cep135, which exceeds the distal end of PACT signal, shows spatial interaction with Cp110. Scale bars, (*a*–*d*) 1 µm.

The primary spermatocytes of *Drosophila* are found in cysts of 16 cells that arise from four rounds of stereotyped divisions of a gonial cell. Each spermatocyte has four centrioles arranged as two V-shaped pairs and each grows about four fold prior to entry into meiosis. We first examined the distribution of Dplp on these centrioles and found it to occupy a region of approximately 360 nm in length (figure 5*a*). This is very similar to the length of the centrioles in spermatogonial cells that undertake the four rounds of mitosis and precede this extended G2 phase. The length of this region of Dplp staining did not appear to change significantly as the centriole lengthened from the very earliest primary spermatocytes to cells immediately prior to meiosis, consistent with a previous report [72]. By contrast, the PACT domain of Dplp (in a line expressing GFP-tagged PACT) was present in a domain immediately within the region of anti-Dplp staining but devoid of the hinge area reflecting the association of GFP-PACT with microtubules (figure 5*a*). This accords with our finding that Dplp lay on the outer face of the short microtubule cylinder of cultured cells. Examination of centrioles throughout the extended G2 showed that indeed primary spermatocytes entered G2 with a 250 nm-long inner cylinder of GFP-PACT surrounded by an outer cylinder of anti-Dplp staining (figure 5*a*). As cysts of primary spermatocytes passed through the 3 day-long G2 phase, this proximal structure (marked by the anti-Dplp staining) increased only from 360 to 540 nm, whereas the cylinder of GFP-PACT grew to a final length of approximately 1400 nm (figure 5*a*). Thus, it seems that the length of the proximal part of the centriole becomes fixed after the mitotic divisions and becomes extended by microtubule growth in the growing stage in such a way that it acquires a restricted set of centriolar antigens.

Sas-4 also occupied the proximal most 360 nm of the mature centriole but lay inside the short Dplp cylinder and thus in the vicinity of the centriolar microtubules in their proximal most part. Cep135, on the other hand, localized to the most inner region of the centriole tube down the full length (compare figure 5*b* and figure 5*c*). Asl co-localized with Dplp at the proximal part but also extended the full length of the centriole in a repetitive punctate pattern (figure 5*c*). Thus, in this proximal part of the mature spermatocyte centriole, Sas-4, Cep135, Dplp and Asl have a similar spatial relationship as we found in the centrioles of cultured cells. However, they differ in the extent to which they extend along the length of the mature centriole. It is perhaps surprising that Sas-4 is not associated with the full length of the centriole but only with the proximal part because centriole elongation is associated with Sas-4 function [42–44]. Sas-4 is also a known partner of Asl [26], and yet its distribution in mature spermatocyte centrioles suggests that Sas-4 is unlikely to be physically associated with Asl in the centrioles' mid and distal parts. We also found that the distal most part of the centriole was capped by a Cp110 plug just as we found in the shorter centrioles of cultured cells (figure 5*d*). Curiously, Cep135 extended beyond the region occupied by PACT right up to the position of the Cp110 cap itself. We have no explanation for this aspect of the distal structure of the spermatocyte centriole. The intensity of Cp110 staining diminished during the growing phase and was totally absent in centrioles greater than 1000 nm in length. Thus, mature spermatocyte centrioles more resemble ‘internal cilia’ in that they lack the capping protein associated with centrioles of other cell types.

**Figure 6.**
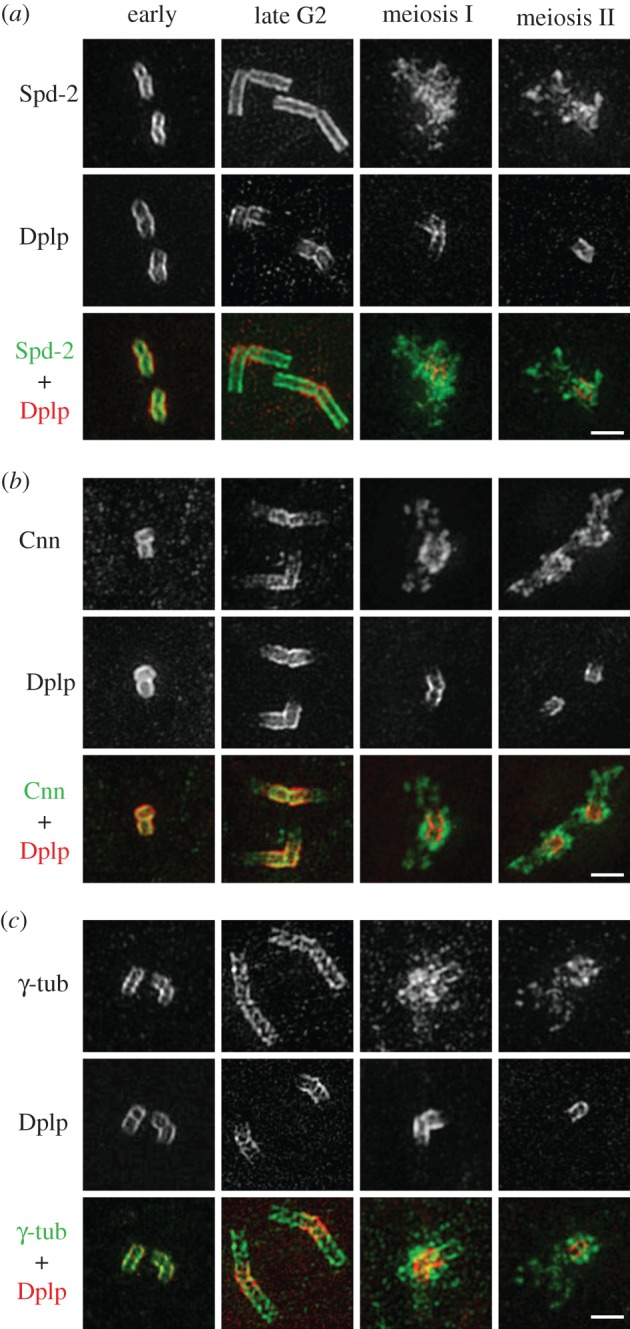
Localization of PCM proteins during development of primary spermatocytes. (*a*–*c*) Spermatocyte centrioles in early and late G2 and in meiosis I and meiosis II stained to reveal Spd-2, Cnn, and γ-tubulin (green) and Dplp (red). In early and late G2 spermatocytes, Spd-2 localizes inside the Dplp tube but extends to the distal part. Cnn and γ-tubulin overlap with Dplp; γ-tubulin also extends along the length of the centriole, while Cnn is predominantly but not exclusively at the proximal end. During meiosis I and II, Spd-2, Cnn and γ-tubulin all expand into the PCM; Cnn is more closely associated with the centriole core than Spd-2 and γ-tubulin. Scale bars, (*a*–*c*) 1 µm.

As the events of centrosome maturation upon entry into meiosis are poorly characterized in *Drosophila*, we examined the meiotic distributions of Spd-2, Cnn and γ-tubulin, which we had found to make major contributions to the PCM in mitotic cells in culture. In both immature and mature spermatocytes, Spd-2 localized inside the Dplp tube and Cnn and γ-tubulin overlapped with it (figure 6). As centrioles grew, anti-Cnn staining was retained predominantly at the proximal ends but extended weakly down the length of the centriole (figure 6*b*). By contrast, strong anti-Spd-2 and anti-γ-tubulin staining extended along the full length of the centriole microtubules, with anti-γ-tubulin staining having a more punctate distribution (figure 6*a*,*c*). The association of Cnn with the proximal part was largely diminished during meiosis I and II, when it expanded into a broad pericentriolar region but remained largely in the vicinity of the centriole cores and proximal ends (figure 6*b*). The association of Spd-2 and γ-tubulin with the entirety of the centriole length was almost entirely lost during meiosis I and II, when the space occupied by the two proteins expanded to generate a cloud of PCM (figure 6*a*,*c*). Thus, the transition of these three molecules from having a physically close association with centrioles towards the end of G2 to become truly pericentriolar in M phase is broadly similar in both cultured cells and spermatocytes.

## 4. Concluding remarks

In both cultured cells and in primary spermatocytes, it is clear that the proteins that are the major constituents of the PCM first associate with the centriolar microtubules before the PCM is formed upon entry into M phase. In cultured cells, these are Spd-2 found in zone II that is occupied by the centriolar microtubules, and Cnn and γ-tubulin that occupy zone III, an immediately outlying layer. Sas-4, implicated in PCM recruitment [26,50], also lies in zone II, suggesting that it could be responsible for Spd-2, and hence γ-tubulin recruitment. However, arguing against this is the finding that although Spd-2 and Sas-4 occupy similar layers in the proximal part of spermatocyte centrioles, only Spd-2 extends throughout the length of the structure. γ-tubulin also extends throughout the centriole but lies mainly in zone III. The presence of Asl and Spd-2 along the whole length of the centriole could accord with the report that Asl is required for Spd-2 to localize to the centrosome [21]. By contrast, the high concentration of Cnn in the proximal most part of zone III could be an indication that its recruitment might be influenced by Sas-4. Dplp, also found mainly in the proximal part of zone III, could also participate in restricting Cnn mainly to this part. A role for Sas-4 in directing PCM formation is indicated by two independent studies [26,50]. In this light, one could also therefore consider the possibility that the proximal part of the spermatocyte centriole provides a seed for PCM formation that then spreads distally and incorporates Spd-2 and γ-tubulin. In this process, Spd-2 is substantially eliminated from spermatocyte centrioles. This contrasts with entry into mitosis in cultured cells, where Spd-2 expands into the PCM while retaining association with zone II of the centriole. Although Polo kinase plays a major role in establishing the PCM, other factors clearly influence how PCM components first associate with different regions of centrioles. The present study identifies spatial restraints that limit the possibilities of molecular partners of PCM components. However, further study is required to define these associations and their dynamics at the molecular level.

## 5. Material and methods

### 5.1. Antibodies

The following primary antibodies were diluted 1 : 500 for use: chicken anti-Dplp [24]; rabbit anti-Asl [26]; rabbit anti-Cp110 [45]; rabbit anti-Spd-2 [36]; rabbit anti-Cnn [22]; mouse anti-γ-tubulin (GTU88, Sigma); mouse anti-α-tubulin (DM1a, Sigma); rabbit anti-phospho-Histone H3 Ser10 (Millipore); mouse anti-phospho-Histone H3 Ser10 (Cell Signaling). Rabbit anti-Cep135 was kindly provided by Timothy Megraw [64]; rabbit anti-Sas-4 by Jordan Raff [61]; and mouse anti-Sas-4 by Tomer Avidor-Reiss (1 : 50) [50]. Secondary antibodies conjugated with Alexa Fluor 488, 594 or 405, diluted 1 : 500 for cells and 1 : 200 for testes, were obtained from Invitrogen.

### 5.2. RNAi depletion, inhibitor treatment and transient transfection

Double-stranded RNA (dsRNA) against Polo, Cnn, Spd-2, Asl, Dplp and Sas-4 were synthesized from templates of the appropriate cDNA clones using the T7 RiboMAX Express RNAi System (Promega) and the following primers:

GST-F: 5′- TAATACGACTCACTATAGGGAGATTTGTATGAGCGCGATGAAG -3′
GST-R: 5′- TAATACGACTCACTATAGGGAGAAACCAGATCCGATTTTGGAG -3′
Polo-F: 5′- TAATACGACTCACTATAGGGAGAGGAGTTCGAATGCCGCTACTACATT -3′
Polo-R: 5′- TAATACGACTCACTATAGGGAGATCAGACAAGAGCTGGGCAAGAACAT -3′
Cnn-F: 5′- TAATACGACTCACTATAGGGAGAACCTCCAGGCGGCGGCAACT -3′
Cnn-R: 5′- TAATACGACTCACTATAGGGAGATGGCTCGAGCGGCATCCTT -3′
Spd-2-F: 5′- TAATACGACTCACTATAGGGAGAGTCGCGTTCCAGCCAAGCAAAGA -3′
Spd-2-R: 5′- TAATACGACTCACTATAGGGAGATCCCCCACCTCCGTTAAGACTCAG -3′
Dplp-F: 5′- TAATACGACTCACTATAGGGAGACTTCTAAGGAAGCCGTGG -3′
Dplp-R: 5′- TAATACGACTCACTATAGGGAGATTCCAGCACCTTGGACTC -3′
Sas-4-F: 5′- TAATACGACTCACTATAGGGAGAATGCAGGAGGCTGGCGAAAGTCC -3′
Sas-4-R: 5′- TAATACGACTCACTATAGGGAGAGGAGGCTTCATCATCGGCATGAG -3′
Asl-F: 5′- TAATACGACTCACTATAGGGAGAATGAACACGCCAGGTATAAG -3′
Asl-R: 5′- TAATACGACTCACTATAGGGAGATATTGGAGCACGTCTCTTT -3′

D.Mel-2 cells were transfected with dsRNA as described [45]. For repeated rounds of depletion, cells were harvested every 3 days and re-submitted to the same transfection protocol. The total numbers of rounds of RNAi used to achieve depletion were: Spd-2 and Cnn, three rounds of 3 days; Asl, Sas-4 and Dplp, two rounds; and Polo, 1 round.

To inhibit Polo kinase, cells were plated for 1 h and then treated with 1 μM BI2536 and incubated overnight prior to analysis.

To express the GFP-tagged constructs, GFP-Plk4, Sas-4-GFP or GFP-Polo, cells were transfected using the X-tremeGENE HP DNA Transfection Reagent (Roche) following the manufacturer's protocol. Cells were collected and subjected to immunostaining after 24 h.

### 5.3. Sample preparation

D.Mel-2 cells were plated on concanavalin A (Sigma)-coated coverslips (#1.5, 0.17 mm thick) 3 h prior to fixation. Cells were washed once with phosphate-buffered saline (PBS) and fixed with pre-cooled methanol for 6 min at −20°C. After rehydration in PBS, the cells were incubated with the primary antibody overnight at 4°C, and subsequently washed and incubated with the secondary antibody for 45 min at room temperature. Coverslips were mounted onto slides using VECTASHIELD mounting medium with or without 4′,6-diamidino-2-phenylindole (DAPI; VECTOR laboratories). To view anti-α-tubulin-stained centrioles, cells were first treated with 1 µg ml^−1^ colchicine (Sigma) overnight, pre-extracted with 0.5 per cent Triton X-100/PBS for 2 min and fixed with pre-cooled methanol for 10 min at −20°C.

Testes from late stage pupae were dissected and washed in PBS, transferred to PBS–5 per cent glycerol and squashed. After snap-freezing in liquid nitrogen, testes on slides were fixed in cold methanol for 10 min, rehydrated in PBS–0.5 per cent Triton X-100 for 1 min and rinsed in PBS for 10 min. Primary antibodies and secondary antibodies were each applied overnight at 4°C and coverslips were mounted onto slides using VECTASHIELD mounting medium with or without DAPI.

### 5.4. Structured illumination microscopy and data processing

Images were acquired using DeltaVision OMX 3D-SIM System V3 (Applied Precision). All data were captured using an Olympus 100x 1.4NA oil objective, 488 nm/593 nm/405 nm laser illumination and standard excitation and emission filter sets. ZEISS immersion oils 1.512, 1.513 or 1.514 were applied to obtain the best resolution. Sections were acquired at 0.125 μm *z* steps, and raw three-phase images were reconstructed in three-dimensional and re-aligned by softWoRx v. 5.0.0 software (Applied Precision). To obtain top views of the centrioles, reconstructed images were processed for maximum-intensity projections; to generate side views, reconstructed images were rotated 90°C around the *x-* or *y*-axis, both with softWoRx.

To measure centriole diameters given in figure 2, the line scan and plot profiles of ImageJ were applied to the top views of centrioles. The distance between peak intensity and the centre of the centriole ring was defined as *b/2*, while the distance from the outer point of minimum intensity to the centre as *a/2.* For each protein, 20 centrioles were measured and s.d. (standard deviation) determined. To analyse distributions of proteins detected in different channels (figure 1*a–c*), RGB-merged files were subjected to line scan and RGB profiler, and different diameters *b* compared.

To address the hierarchy of PCM recruitment (figure 4), the fluorescence intensities of γ-tubulin, Spd-2, Cnn and Dplp were determined on maximum-intensity projections by ImageJ. Each value was measured in a constant region centred on the centrosome, and the background signal from an adjacent identically sized area subtracted. The value of the target protein was then normalized to that of Dplp. Finally, for the control group in each experiment, this value was arbitrarily set to 10. The overall data were plotted as scatter dot plots showing mean, s.e.m. and *p*-value using Prism v. 5. Each s.e.m. was achieved from at least 30 centrosomes and the *p*-values were obtained by Student's *t*-test. To exclusively measure the fluorescence intensity of PCM Spd-2, a circular mask was applied to subtract the centriolar cylinder before taking similar measurements (figure 4*d*).

To obtain the surface rendering of centrosomes in figure 2*a–c*, the Huygens Essential tool was applied on reconstructed stacks and surface rendering carried out. To measure the volume of Cnn in figure 4*f*, the Huygens Object Analyser advanced tool was applied, and for all datasets, the threshold was set to 20 per cent before the volume was calculated.

## 6. Acknowledgements

We are very grateful to the Cancer Research UK for support of this work and to the Royal Society for a Newton Post-Doctoral Fellowship to J.F. We also thank the Wellcome Trust for a grant enabling the purchase of the OMX microscope. Thanks are also due to Nicola Lawrence and Alex Sossick for assistance with microscopy. We very much appreciate the advice of Hélène Rangone, Matthew Savoian and Tetsuya Takeda in these experiments and the helpful comments of Paula Coelho and Nick Dzhindzhev on the manuscript. Antibodies were very kindly provided by Jordan Raff (rabbit anti-Sas-4), Tomer Avidor-Reiss (mouse anti-Sas-4), Tim Megraw (anti-Cep135), and fly lines by Mónica Bettencourt-Dias (*polyubiquitin GFP-Cep135/CyO*); Jordan Raff (*polyubiquitin GFP-PACT/CyO*).
